# Structural Basis for pH-mediated Regulation of F-actin Severing by Gelsolin Domain 1

**DOI:** 10.1038/srep45230

**Published:** 2017-03-28

**Authors:** Jing-song Fan, Honzhen Goh, Ke Ding, Bo Xue, Robert C. Robinson, Daiwen Yang

**Affiliations:** 1 Department of Biological Sciences, National University of Singapore, 14 Science Drive 4, 117543, Singapore; 2 Institute of Molecular and Cell Biology, A*STAR (Agency for Science, Technology and Research), Singapore; 3 Department of Biochemistry, National University of Singapore, Singapore; 4 NTU Institute of Structural Biology, Nanyang Technological University, 59 Nanyang Drive, 636921, Singapore

## Abstract

Six-domain gelsolin regulates actin structural dynamics through its abilities to sever, cap and uncap F-actin. These activities are modulated by various cellular parameters like Ca^2+^ and pH. Until now, only the molecular activation mechanism of gelsolin by Ca^2+^ has been understood relatively well. The fragment comprising the first domain and six residues from the linker region into the second domain has been shown to be similar to the full-length protein in F-actin severing activity in the absence of Ca^2+^ at pH 5. To understand how this gelsolin fragment is activated for F-actin severing by lowering pH, we solved its NMR structures at both pH 7.3 and 5 in the absence of Ca^2+^ and measured the pKa values of acidic amino acid residues and histidine residues. The overall structure and dynamics of the fragment are not affected significantly by pH. Nevertheless, local structural changes caused by protonation of His29 and Asp109 result in the activation on lowering the pH, and protonation of His151 directly effects filament binding since it resides in the gelsolin/actin interface. Mutagenesis studies support that His29, Asp109 and His151 play important roles in the pH-dependent severing activity of the gelsolin fragment.

Gelsolin is an actin-regulatory protein comprising six domains. It exists in two major forms: cytoplasmic and plasma gelsolin, and the latter contains an extra signal peptide (24 amino acids)^1^,^2^. The cytoplasmic form plays a critical role in remodelling the actin cytoskeleton during cell movement via severing, capping, and uncapping actin filaments. The remodelling is regulated by Ca^2+^, pH, and phosphatidylinositol-4,5-bisphosphate (PIP2)^1^,^3^. Plasma gelsolin is highly abundant in human extracellular fluids and primarily responsible for elimination of toxic actin filaments from the bloodstream that are released by damaged cells^4^,^5^. Decreased levels of gelsolin have been found in patients with various pathological diseases, and an inverse correlation has been observed between circulating gelsolin levels and likelihood of mortality^5^,^6^. Moreover, repletion of plasma gelsolin significantly improves survival in animal models^7^. Thus gelsolin replacement may be considered as a potential therapy^8^,^9^. Besides full-length gelsolin, some gelsolin fragments have F-actin severing activity and also have a therapeutic effect in disease conditions accompanying cell injury^8^,^10^.

Recent truncation studies have shown that the minimal gelsolin fragment (residues 28–161) consisting of domain 1 (G1) and the linker between domains G1 and G2 depolymerizes F-actin more efficiently than the full-length protein and other truncation mutants *in vitro* and in animal models^10^. This minimal fragment, denoted as G1+ hereafter, is nearly identical to the construct (residues 25–160) used previously for structural determination in complex with actin^11^. Interestingly, the F-actin depolymerization activity of G1+ is dependent on Ca^2+^ and pH, similar to the full-length protein but different from the N-terminal half including domains G1-G3. Previous reports have demonstrated that gelsolin requires no Ca^2+^ for *in vitro* binding to G-actin, nucleating F-actin formation, and severing F-actin filaments at a pH less than 6.0^12^,^13^. According to recent small angle X-ray scattering (SAXS) data, lowering pH from 8 to 5 induces the increase of the gyration radii of gelsolin and G1+ in the absence of Ca^2+^ by 9.7% and 11.6%, respectively^10^,^14^. For the full-length gelsolin, the gyration radius starts to increase at about pH 6^14^, correlating with its activity dependence on pH^12^,^13^. Although it is unclear at which pH the gyration radius of G1+ starts to change, G1+ is inactive at pH 7–8 and becomes partially active at pH 6 in the absence of Ca^2+^ 10. SAXS-based structure reconstructions suggested a pH-activated “open” state of gelsolin in which G1 is detached from domain G3 and domains G2-G6 still retain the inactive-like structure. This state is different from the Ca^2+^-activated state in which the C-terminal latch is detached from domain G2 and the other two latches between domains G1 and G3 and between domains G4 and G6 are also released^15^,^16^. Due to the unavailability of atomic resolution structure of gelsolin at pHs lower than 6 in the absence of ligands, the exact activation mechanism by pH is still unclear. In addition, G1+ was proposed to assume a “closed” state in which the linker between domains G1 and G2 seems to be in contact with G1 in the absence of Ca^2+^ at pH 8, but to adopt an “open” state in which the linker may extend away from G1 at pH 5^10^. According to the activity dependence on pH, the Ca^2+^-free G1+ should exist in a “closed” state at a pH larger than 7. The proposed “open” conformation is supported by the structure of Ca^2+^-bound G1+ in complex with G-actin. Nevertheless, lack of the structure in the absence of Ca^2+^ at low pH prevents understanding the mechanism of G1+’s action.

In order to address how G1+ is activated by lowering pH, we solved its structures at pH 7.3 and 5, and measured the pKa values of Asp, Glu, and His residues by nuclear magnetic resonance (NMR) spectroscopy. The overall structures at the two pH values are very similar, but local structural differences lead to an active and an inactive form. The structural changes are caused largely by protonation of His29 and moderately by protonation of Asp109 whose pKa values are close to 6, while protonation of His151 directly regulates the G1+/actin interaction. Our mutagenesis data also support that His29 dominates the inhibition of F-actin severing activity by G1+ at pH 7.3. This inhibition is overcome by protonation of His29 and released by removal of the sidechain (His29Ala mutation).

## Results

**Figure 1:**
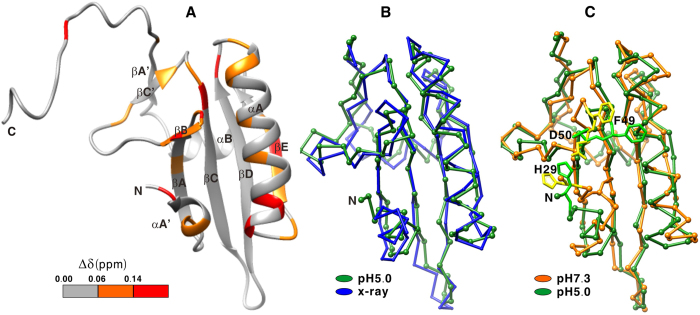
Lowest energy structure of G1+ solved at pH 5.0 (**A**), comparison of the calcium-free structure at pH 5.0 with the X-ray structure in complex with actin and calcium (PDB: 1P8Z) (**B**), and comparison of calcium-free structures at pH 5.0 and 7.3 (**C**). (**A**) Ribbon representation with highlighted residues (red) that displayed large (Δδ ≥ Δδ_av_ + std) and moderate (Δδ_av_ < Δδ < Δδ_av_ + std) chemical shift perturbations by pH. The average chemical shift difference between pH 5.9 and 7.3 over all available residues (Δδ_av_) was 0.06 ppm, while the standard deviation of chemical shift differences was 0.08 ppm. In (**B**) and (**C**), the disordered C-terminal tail (145–161) are not displayed. In (**C**), His29, Phe49, and Asp50 (including sidechain and backbone) at pH 5.0 and 7.3 are highlighted in light green and yellow sticks, respectively.

At pH 5.0, 130 out of 134 residues in G1+ (Glu28-Arg161) were unambiguously assigned in backbone resonances, while ~95% of the sidechain resonances were assigned. Unassigned residues were Glu28, Glu32, Phe62, and His151. The solution structure was determined using 972 distance restraints and 88 dihedral angle restraints (Tab. S1) which were derived from nuclear Overhauser effects (NOEs) and chemical shifts, respectively. The average RMSDs of the 20 lowest energy structures are 0.87 ± 0.12 and 1.64 ± 0.12 Å for backbone and all heavy atoms in structural regions, respectively. G1+ is arranged around a central β–sheet with four anti-parallel strands (βA, 40–47; βB, 50–52; βC, 67–75; βD, 81–88) and one parallel strand (βE, 117–122) (Fig. 1A). On one side of the sheet there is a long helix (αA, 95–112) which is approximately parallel to the sheet and a 3_10_-helix (αA′, 31–33), while on the other side there are a short 2-stranded parallel β-sheet (βA′, 140–142; βC′, 62–64) and a short helix (αB, 128–133) which is approximately perpendicular to the sheet. The C-terminal tail (145–161) is disordered. As no NOEs were found between this tail and the rest of G1+, the orientation of the tail was not defined. The overall solution structure is similar to the structure of G1+ in complex with G-actin and Ca^2+^ solved by X-ray crystallography (Fig. 1B). The RMSD of backbone atoms in the resolved regions between the NMR and X-ray structures is 1.2 Å. The obvious differences lie in βB, βC’, loops and the C-terminal tail.

At pH 7.3, 13 residues (Glu28, Arg77, Asn78, Gln95, Lys135, Gly144 - His151) were not assigned because their backbone amides were undetectable. Most unassigned residues are located in the C-terminal disordered region. In total, 908 distance and 81 dihedral restraints were obtained. With these restraints, the structure was determined. The overall structures at pH 5 and 7.3 are similar. The major differences are located in αA′, βB, and βA-βB loop (Fig. 1C). In addition, the orientation of αA relative to the β-sheet is slightly altered. At pH 5, the sidechain of His29 is close to the sidechain of Asp50 at βB in most of the 20 lowest energy structures. At pH 7.3, however, it moves away from the sidechain of Asp50. Moreover, the locations of Phe49 and Asp50 sidechains at the two pHs are very different (Fig. 1C).

**Figure 2:**
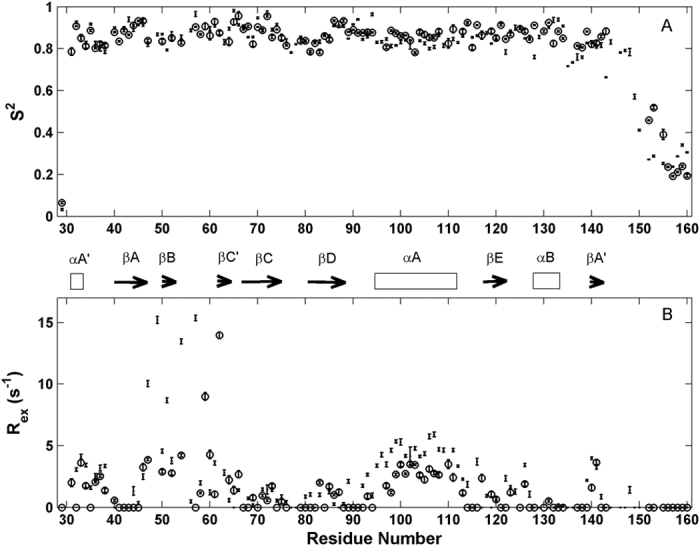
Order parameters (S^2^) and conformational exchange contribution to transverse relaxation rates (R_ex_) of G1 + at pH 5.0 (·) and pH 7.3 (o). The locations of secondary structure elements are indicated on top of panel B. The errors associated with each measurement are indicated by vertical bars.

To examine if the C-terminal region is more restricted in motion or more rigid at pH 7.3 than at pH 5.0, we measured the ^15^N relaxation times and heteronuclear NOEs. Order parameters derived from ^15^N relaxation data are shown in Fig. 2A. At pH 5.0, the average order parameter for the region of 32–148 (0.86 ± 0.06, average value of 0.86 and standard deviation value of 0.06) was significantly larger than that for the C-terminal region of 149–160 (0.32 ± 0.10). Moreover, the α helices, β strands, and loops between the regular secondary structure elements displayed similar order parameters. The order parameter of a bond vector reflects its flexibility on ns – ps timescales, which ranges from 0 (fully mobile) to 1 (completely rigid). Therefore, the protein backbone is rather rigid except the C-terminal tail and N-terminal residues Glu28 and His29. The order parameters at pH 7.3 (0.87 ± 0.04 for the region of 31–143 and 0.30 ± 0.13 for the region of 152–160) are very similar to those at pH 5.0 (Fig. 2A), showing that the protein flexibility on ns-ps timescales is not altered significantly by pH.

To investigate if the protein has similar mobility on ms-μs timescales at pH 5.0 and 7.3, we extracted the contributions of conformational exchange to transverse relaxation rates (R_ex_) from the relaxation data. At pH 5.0, several regions displayed R_ex_ values larger than 2 s^−1^, including αA′, loop αA′– βA, loop βA – βB, βB, loop βB – βC′, βC′, αA, and βA′ (Fig. 2B). The result implies that these regions are dynamic on ms – μs timescales or these regions exist in at least two distinct conformational states although they are rigid on ns – ps timescales. The same regions are also dynamic at pH 7.3 since their R_ex_ values at pH 5.0 and pH 7.3 are similar (Fig. 2B). Interestingly, the βC- βD loop is quite rigid on both ns - ps and ms - μs time scales. Its rigidity may be important for G1+’s severing activity since when G1+ binds to an actin protomer in the F-actin, the steric clashes between this loop and the adjacent actin protomer may help to disrupt the actin-actin interactions^17^.

Attempting to obtain more detailed information about conformational exchanges between different states, we performed ^15^N relaxation dispersion experiments. The residues located in the regions without R_ex_ (R_e_ ≈ 0) display no relaxation dispersion (Fig. S1d and h). The residues located in the regions with R_ex_ values larger than 2 s^−1^ exhibit slight relaxation dispersion (Fig. S1a–c and e–g). For example, the difference of relaxation rates for Asn57 at CPMG field strengths of 50 and 1000 Hz was 2.7 s^−1^ although its R_ex_ value derived from the model-free analysis was 15.4 s^−1^. Due to the small dispersion, we were unable to calculate the exchange rates between different conformational states and obtain structural information of the “invisible” states with population less than 10%. If the exchange process was on a millisecond timescale or slower, large ^15^N relaxation dispersion should be observed. Therefore, the conformational exchange in αA′, loop αA′– βA, βB, loop βB – βC′, βC′, αA, and βA′ should occur on a sub-millisecond timescale at both pH 5.0 and pH 7.3.

Phe144-Lys150 were not observable in the HSQC spectrum at pH 7.3, but gave rise to strong signals at pH 5.0. The result indicates that the region around His151 undergoes conformational exchange on ms-μs timescales only at pH 7.3, implying this region has weak interactions with other regions of the protein at high pH. Due to this interaction, the gyration radius of G1+ may be smaller at high pH (≥7) than that at low pH (≤6) as the previous SAXS data imply^10^. Unfortunately, we could not determine which residues are in contact with the region around His151.

Except for Glu28 and Glu31, sidechain ^13^CO spins of other Glu and all Asp residues were assigned at both pH 5.0 and 7.3 using 3D HCACO spectra. The sidechain of Glu31 was assigned only at pH 7.3. The ^1^H_δ_ and ^13^C_δ_ spins of all His sidechains were assigned from the 4D NOESY. The ^1^H_β_-^13^CO correlations of Asp and ^1^H_γ_-^13^CO correlations of assigned Glu were resolved at pH 3.5 (Fig. S2). The dependences of Asp and Glu ^13^CO chemical shifts on pH are shown in Fig. S3 and S4, respectively. The dependences of His ^1^H_δ_ or ^13^C_δ_ chemical shifts on pH are given in Fig. S5. Through fitting the data of each residue shown in Fig. S3–5 to eq. (1), we obtained the pKa values. The results are summarized in Table 1. The fitting errors of pKa values were about 0.2. Because the protein started to unfolded at a pH equal to or lower than 3, we were unable to determine the exact pKa for the residues with pKa values smaller than 3.5. Glu31 gave rise to very weak HSQC signals at pHs lower than 7.3 and its ^1^H_γ_-^13^CO correlation was too weak to be detected at pHs below 6. Thus the pKa of Glu31 was not measured. The pKa values of Asp, Glu, and His in the absence of interactions (e.g., in the GlyXGly tripeptide) are 3.9, 4.3 and 6.4, respectively^18^. Thus Asp50 and Asp109 have significantly upshifted pKa values, while E47, E92, E97, E121, His86, and His119 have significantly downshifted pKa values.

**Figure 3:**
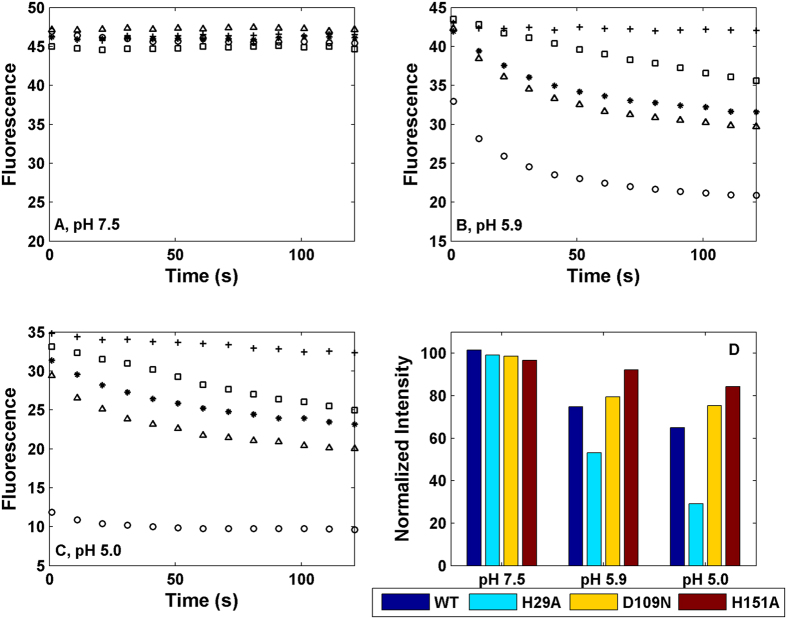
Changes of fluorescence intensities of fluorescently-labeled F-actin in buffer alone (+), WT G1+ (Δ), His29Ala (o), Asp109Asn (*), and His151Ala (o) at pH 7.5 (**A**), 5.9 (**B**), and 5.0 (**C**). (**D**). The relative intensities at a time of 60 seconds, which were normalized relative to intensities for the control samples. The uncertainties for the intensities at 60 s were about 5% based on three independent measurements.

A recent study shows that G1+ has no F-actin severing activity at pH 7.0 in the absence of Ca^2+^, but it becomes partially active at pH 6.0 and the activity is further elevated at pH 5.0^10^. This conclusion was confirmed here by an F-actin depolymerization assay (Fig. 3), which monitors the combined effects of filament severing and monomer sequestration. Loss of filaments on short time scales is interpreted to have a significant severing element to the depolymerization. The effect of pH on protein function often results from protonation or deprotonation of basic or/and acidic residues. Our pKa data indicate that only three residues (His29, Asp109, and His151) can have significant changes in protonation in moving from pH 7.3 to 5. To understand which residues contribute to the pH effect, we prepared three single point mutants: His29Ala, Asp109Asn, and His151Ala. The F-actin severing activities for the three mutants are shown in Fig. 3. At pH 7.5, the wild type G1+ and its three mutants behaved identically and displayed nearly no decay in fluorescence intensities of pyrene-labeled F-actin within 120 seconds (Fig. 3A,D). The result indicates that G1+ and its mutants have no severing capability at pH 7.5 in the absence of Ca^2+^. At pH 5.9, the wild-type G1+ depolymerized F-actin at a significantly slower rate than the His29Ala mutant, but at a significantly higher rate than the His151Ala, while at a slightly quicker rate than the Asp109Asn mutant (Fig. 3B). At pH 5.0, similar trends were observed though the depolymerization for all the samples were further enhanced (Fig. 3C,D). The results suggest that His29 may be critical to the inhibition of severing, which is somewhat overcome by protonation and removed by mutation to alanine. However, a second element of protonation of Asp109 and His151 is also required, since His29Ala is not active at pH 7.5.

To understand the structural basis of the pH-induced F-actin depolymerization, we used 2D ^1^H-^15^N HSQC experiments to probe chemical shift perturbation by pH. Several residues displayed large and moderate chemical shift changes when the pH was reduced from 7.3 to 5.9 (Figs S6 and 7), indicating changes in their chemical environments. Some of these residues are located in the vicinities of His29 (His29, αA′: Phe32-Lys34, βB: Asp50-Val52), Asp109 (αA: Thr105, Asp109, Asp110, Leu112; αA-βE loop: Gly114, Arg115; βE: Val117), His119 (βE: Gln118-Arg120), and His151 (Val152). The rest are located in the regions distant from these four charged residues but are directly or indirectly associated with βB (βA-βB loop: Glu47-Phe49, βB-βC′ loop: Tyr59 and Gly60, βC′: Phe63, βC′-βC loop: Asp66, βC: Ala67; βD-αA loop: Gly90, Ser94; N-terminal end of αA: Asp96, Glu97) (Fig. 1A). αA’, βA-βB loop, βB, and N- and C-terminal ends of αA display differences in local structure at pH 7.3 and 5.0 (Fig. 1C) and also have significant chemical shift perturbations by pH (Fig. 1A), indicating that protonation of residues alters local structures. If the protonation of an ionizable group does not affect protein structure, the chemical shift perturbation is only limited to the nuclei close to the ionizable group^19^. Many residues distant from His29, Asp109 and His151 exhibit significant chemical shift perturbations, demonstrating that protonation of these residues can induce conformational changes. Previous studies have also shown that changing the charge of amino acid sidechains can drive changes in protein conformation and function^20^,^21^.

## Discussion

**Figure 4:**
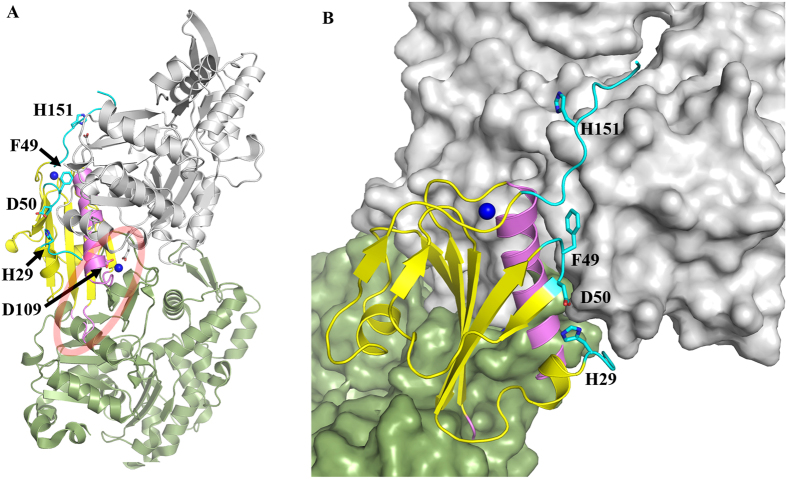
Location of critical G1+ residues in relation to F-actin binding. (**A**) The structure of G1+/G-actin was superimposed onto a protomer in the structure of F-actin. (**B**) A close up of the G1+/actin interface. Only one F-actin protomer (green) is shown together with the G-actin (grey). G1+ is painted yellow with regions colored in cyan showing G1+ interactions within the model which have no steric clash with F-actin, whereas regions highlighted in pink have steric clashes highlighted by the red ellipse. Ca^2+^ ions are indicated by blue balls.

The hydrogen ion (H+) can be considered as the smallest ligand. Its association to, and dissociation from, acidic and basic residues of proteins play important roles in protein folding, enzymatic activity, and cellular processes^20^. The protonation state of sidechains of acidic and basic residues depends on their pKa values and the environmental pH. The standard pKa values of Asp, Glu, His, Arg and Lys are 3.9, 4.3, 6.4, 13.9, and 10.3, respectively, when their sidechains are isolated without any interactions with their proximal residues^18^. Due to charge-charge interactions, hydrogen bonding, and desolvation effects, the pKa values of the acidic and basic residues of a protein often deviate from the standard values^22^. There are 16 acidic residues (7 Asp and 9 Glu) and 18 basic residues (4 His, 5 Arg and 9 Lys) in G1+. Except for Glu28 and Glu31, we measured the pKas of 14 acidic residues plus all 4 histidine residues. The pKa values of Asp50 and Asp109 are increased significantly above the free amino acids, indicating that their charged states are less favorable than the uncharged states under moderately acidic conditions. The carboxyl group of Asp109 is next to Asp110 and partially buried in a hydrophobic environment (formed by Val106, Gly114, and Ala116), which together elevate its pKa value to 5.5. Asp109 is the sidechain that can coordinate calcium in the G1+/actin interface (Fig. 4A). The sidechain of Asp50 located in βB is exposed to solvent but contacts Val52 on the adjacent strand βA and His29 (pKa 5.9) in the N-terminus. At neutral pH, these will provide a hydrophobic environment that upshifts the pKa of Asp50. Its pKa is further elevated by the negatively charged Glu47 (pKa < 3.5) since the sidechains of Asp50 and Glu47 are close to each other. In the G1+/actin structure Asp50 lies in the interface contacting actin residue Ser350. Glu47 and Arg45 are on the same strand and their attractive charge-charge interaction downshifts the pKa of Glu47. Asp84, Asp96, Glu97, Glu121 and Glu126 have significantly reduced pKa values due to charge-charge interactions with their respective proximal positively charged residues. His29’s pKa is downshifted, indicating that its sidechain favors an uncharged state and likely has hydrophobic interactions with its surroundings. The sidechain of His29 is exposed to solvent in most of the calculated structures at pH 7.3 (Fig. 1C), but the sidechain lies on the hydrophobic core centred on Leu51 of G1+ in some structures. This structural uncertainty may result from insufficient distance restraints or/and intrinsic mobility. The downshifted pKa suggests that His29’s sidechain is more likely to be close to the sidechain of Leu51. The significant chemical shift perturbation at Leu51 upon pH change from 7.3 to 5.9 (Fig. S7) indicates that Leu51 is affected by His29 or there are interactions between His29 and Leu51 sidechains. As mentioned earlier His29 also interacts with Asp50, which itself has an elevated pKa, both of which lie at the G1+/actin interface (Fig. 4). The sidechains of His86 and His119 are largely buried and positioned side-by-side, leading to significantly downshifted pKa values. His151 is located in a disordered region. Thus, its pKa is similar to the standard value. This residue also lies at the G1+/actin interface (Fig. 4).

According to the G1+/actin complex structure, the long helix αA of G1+ inserts into the cleft formed at the interface of G-actin subdomains 1 and 3 through both hydrophobic interactions and H-bonding (Fig. 4)^17^. Besides the helix, Phe49 and Asp50 of G1+ form H-bonds with Ser350 of actin, Phe49 and Phe149 of G1+ form hydrophobic interactions with Ile341, Ile345 and Leu349 of actin^17^. The ligand-free G1+ structure at pH 5.0 is very similar to the ligand-bound structure (Fig. 1B), with the sidechains of Phe49 and Asp50 having almost the same orientations in both of them. Since G1+ is active in severing F-actin at pH 5.0, the structure at pH 5.0 may correspond to an active conformation that readily interacts with F-actin. However, initially the long helix αA of G1+ will not be able to approach its binding site on F-actin since that region is occupied by the D-loop from the next actin in the filament. This suggests that the first contact with the filament will be through the regions highlighted in cyan in Fig. 4, after which the long helix αA will compete with the next actin for its binding site.

Although the overall structures of ligand-free G1+ at pH 7.3 and 5.0 are very similar, three regions are different: 1) N-terminal region (Glu28-Glu38) including αA′ and the αA′ - βA loop, 2) the βA - βB loop, and 3) the C-terminal end of the long helix (Fig. 1C) (Fig. 4, cyan). Thus the structural changes and changes in charge states of acidic and basic residues in these three regions may make the initial docking of G1+ to F-actin unfavorable at pH 7.3. Because G1+ is inactive at high pH (≥7)^10^, the structure at pH 7.3 may represent an inactive conformation which has low affinity to F-actin.

Because G1+ shows obvious severing activity at about pH 6, structural changes from the inactive to the active form should result from the protonation of the residues with pKa values close to 6. The standard pKa values of Arg and Lys are above 10.3. The pKa values of these two types of residues in G1+ should be significantly larger than 6 since they are not buried in hydrophobic cores and do not form positively charged clusters. So, only Asp109, His29 and H151 have pKa values close to 6.0.

According to chemical shift perturbation by changing pH from 7.3 to 5.9 (Fig. 1A, Fig. S7), structural changes are mainly located in the vicinity of His29, Asp109, His119, and His151 and as well the regions associated with βB. Hence these four residues may contribute to the pH-induced F-actin depolymerization activity. His151 is in a disordered tail and its protonation cannot cause distal structural changes. Nevertheless, the His151Ala mutant has lower F-actin depolymerization activity than the WT protein at pH 5.9 and 5.0. According to the G1/actin complex structure, His151 is close to Asp25 of actin (Fig. 4). Substitution of His151 with Ala151 may reduce G1+/actin interaction and thus results in reduction of the activity. The sidechain of His119 in the middle of βE faces αB instead of αA and the orientation change of this sidechain does not affect αA and βB. In addition, the pKa of His119 is 4.3. Therefore, His119 should not contribute to the observed effect of pH on the activity.

His29 sidechain is deprotonated and exists in an uncharged form at pH 7.3, and can form hydrophobic interaction with Leu51 based on the inactive structure and the chemical shift perturbation (Fig. S7). It will have an unfavourable interaction with Asp50 at high pH. At low pH (5.0), His29 sidechain is mainly protonated as a positively charged form, and is not favorable to a hydrophobic environment. This positively charged sidechain will then have an attractive interaction with the negatively charged Asp50. Thus protonation of His29 controls the relative positioning of two actin binding regions, the N-terminus and the βA-βB loop, resulting in an active conformation (Figs 1C and 4). Structural changes in βB can further affect its associated regions since the five strands βA – βE form a sheet. The structural changes are supported by the chemical shift perturbation by pH (Fig. 1A and Fig. S7). Therefore, protonation of His29 is critical to the conformational conversion from an inactive state to an active state.

His29 is about 50% and 90% protonated at pH 5.9 and 5.0, respectively, which may shift the equilibrium of the active and inactive states. Mutation of His29 to Ala further drives the G1+ conformation towards the active state, resulting in higher severing activity. This suggests that the non-protonated form of His29 is an inhibitor of F-actin recognition, protonation of this residue partially removes the inhibition, whereas shortening the sidechain abolishes the inhibition. If His29 were the only contributor to the pH-induced severing activity of G1+, the His29Ala mutant would be pH-independent and remain active at pH 7.5. On the contrary, the severing data indicate that other residues contribute to the pH-regulation of severing activity. Protonation of His151 will enhance actin binding by the positively charged region (Fig. 4).

pH-induced structural changes in the region around Asp109 may also contribute to the effect observed. In the structure of G1+/actin/Ca^2+^ complex, the sidechain of Asp109 in G1+ together with the sidechain of Glu167 in G-actin is bound to Ca^2+^, and Asp110 and Gln107 of G1+ are also involved in interactions with actin through hydrogen bonding. In F-actin, Glu167 of actin can form a salt bridge with Lys61 in the adjacent actin protomer in the absence of Ca^2+ ^23. This interaction might contribute to the pH-dependent stability of F-actin. Indeed, F-actin depolymerization in the control samples was more significant at lower pH values (Fig. 3), indicating F-actin is less stable at lower pH. The Asp109Asn mutant, which is an analogue of the protonated form of Asp109, is just slightly less active than the WT G1+, indicating that the effect of Asp109 protonation on the severing activity is limited. This is not surprising, since Asp109 interacting residue on actin (Glu167) is only accessible after severing has occurred (Fig. 4). Taken together, His29 plays a dominant role in the pH regulated activity, most probably by controlling the relative positioning of two actin-binding regions, namely the N-terminus and the βA-βB loop. Protonation of His151 will directly aid interaction with F-actin, and protonation of Asp109 will stabilize the severed complex and perhaps contribute slightly to activation, since the structure around this residue is perturbed by pH.

Actin cytoskeleton assemblies and cell movement can be regulated by intracellular pH. The regulation is likely achieved through a number of pH sensing actin-regulatory proteins such as talin, cofilin, twinfilin, gelsolin, and adseverin^20^. For talin which has a higher affinity to F-actin at pH 6.5 than at pH 7.5, His2418 with a slightly upshifted pKa has been shown to play a key role in the pH-dependent actin binding^21^. Protonation of this residue has been suggested to modulate conformation and dynamics of distal residues in the actin binding site. Similarly, the protonation of His29 in G1+ induces conformational changes of distal residues in the binding site (βA-βB loop) and in turn regulates the severing activity by pH. Several acidic residues may also play a role together with His2418 in talin since their predicted pKa values are upshifted, but their exact role is unknown due to the unavailability of experimentally determined pKas. In this present study with measurements of pKa values of most acidic residues, we have demonstrated that His29 and His151 in G1+ participate in the pH-mediated severing of actin filament, and that Asp109 likely plays a role in stabilizing the severed complex. Thus, multiple residues of an actin binding protein can be responsible for regulation of actin dynamics through pH change. Besides talin, cofilin has been studied in terms of pH-sensing^24^. Based on molecular dynamics simulations, protonation of His133 has been proposed to modulate the binding of cofilin to actin at the F-site. However, the cofilin His133Ala mutant retained an F-actin severing activity similar to the WT protein, suggesting other titratable residues may contribute to the pH-dependent activity. Interestingly, the WT cofilin is pH-dependent in phosphoinositide binding, but the His133Ala mutant with attenuated phosphoinositide binding is relatively pH insensitive, suggesting that cofilin may regulate actin filament dynamics by pH through the interaction of the charged histidine with phosphoinositide. Further studies on G1+ are required to examine if His29 and His151 are involved in phosphoinositide interactions. Although G1+ is just a fragment of gelsolin, His29, Asp109 and His151 are likely to be important to the full-length gelsolin in the pH-dependent actin dynamics. To further understand how gelsolin is activated by lowering pH, pKa measurements of all histidine residues and acidic residues in the full-length protein are necessary.

## Materials and Methods

### Sample preparation

Recombinant G1+ was cloned into a compatible pET-28a vector with His-tag at the C-terminus of the protein. The plasmid was transformed into BL21(DE3) *E. coli* strain for expression. To obtain ^15^N, ^13^C labeled protein, the culture was allowed to grow in M9 minimal medium containing 1 g/L ^15^N NH_4_Cl and 2 g/L ^13^C labeled glucose at 37 °C until the OD_600nm_ reached 0.6. Induction was done with 1 mM IPTG. Cells were harvested 16 hours later after being left to grow at 16 °C. Subsequently the cells were lysed by sonication. The supernatant obtained after high speed centrifugation was applied onto Ni^2+^-NTA column for purification. The protein was further purified with a gel filtration column Superdex 75. For the sample at pH 7.3, the final buffer used contained 25 mM Tris, 45 mM NaCl, 1 mM EDTA, 1 mM EGTA, and 5% D_2_O. For the sample at pH 5.0, the final buffer contained 25 mM acetate, 45 mM NaCl, 1 mM EDTA, 1 mM EGTA, and 5% D_2_O. Although the previous SAXS experiment on G1+ was done at pH 8, we here carried out experiments for structure determination at pH 7.3. This is because 1). G1+ is not active at pH ≥ 7, thus its conformations should be similar at pH 7.3 and 8, and 2). NMR experimental sensitivity is much lower at pH 8 than at pH 7.3 due to enhanced amide hydrogen exchange by high pH.

### NMR spectroscopy

All NMR experiments were performed on a Bruker Avance 800 MHz spectrometer equipped with a cryo-probe at 25 °C. For resonance assignment and structure determination, 2D HSQC, 3D HNCA, HNCOCA, MQ-(H)CCH-TOCSY^25^ and 4D NOESY^26^,^27^ were recorded on ^13^C,^15^N labeled samples at a protein concentration of 1 mM. Using the same samples, ^15^N relaxation rates R_1_ and R_2_ and heteronuclear ^15^N NOEs were measured by 2D HSQC-based methods. R_1ρ_ values were determined by acquiring 7 points with relaxation delays of 2, 15, 35, 60, 70, and 81 ms using a spin-lock field strength of 1600 Hz. R_1_ values were measured using 7 relaxation delays of 5, 300, 550, 650, 750, 900, and 1100 ms. R_2_ values were calculated from R_1_ and R_1ρ_ values^28^. ^15^N NOEs were measured using two spectra without and with proton saturation with a saturation delay of 5 s and recycle delay of 4 s. Proton saturation was achieved by a train of 120° pulses spaced at 5 ms. ^15^N relaxation dispersion profiles were obtained with a ^1^H-decoupled and phase-cycled CPMG method^29^,^30^ using a constant time relaxation delay of 40 ms and inter-scan delay of 2 s.

A 3D HCACO experiment^31^ was used to obtain H_β_-C_β_-CO correlations for Asp and H_γ_-C_γ_-CO correlations for Glu sidechains. Using these correlations and the assignments of ^1^H_β_, ^13^C_β_, ^1^H_γ_, and ^13^C_γ_, sidechain ^13^CO spins were assigned. Two 3D data sets were acquired, one at pH 7.3 and the other at pH 5. Each data set comprised 640 × 60 × 30 complex points in the ^1^H, ^13^C, and ^13^CO dimensions. It was recorded with 8 scans and an inter-scan delay of 1 s. To monitor the change of ^13^CO chemical shifts with pH, 2D data sets were recorded at a series of pH values using a 2D version of the 3D HCACO experiment. Each 2D data set comprised 640 × 50 complex points in the ^1^H and ^13^CO dimensions. It was recorded with 64 scans, an inter-scan delay of 1 s, resulting in a total time of about 1.8 h. The spectral width in ^13^C dimension was 2414 Hz. To probe the chemical shift changes of ^13^C_δ_ and ^1^H_δ_ of histidine residues, 2D ^1^H-^13^C HSQC data sets were acquired. Each 2D data set comprised 640 × 70 complex points in the ^1^H and ^13^C dimensions and was recorded with 16 scans, an inter-scan delay of 1 s, resulting in a total time of about 45 minutes. The spectral width in ^13^C dimension was 6036 Hz.

### Structure determination

NMR spectra were processed using NMRPipe^32^ and analyzed using Sparky. Backbone and side-chain resonances were assigned using the NOESY-based strategy described previously^26^,^33^. Unambiguous NOEs were obtained from three sub-spectra: ^13^C,^15^N-edited, ^13^C,^13^C-edited, and ^15^N,^15^N-edited 4D NOESY. Ambiguous NOEs were further assigned during iterated structure calculation and refinement. Distance constraints were obtained from the NOEs assigned, while dihedral angle restraints of ϕ and ψ were calculated with TALOS+^34^ using the assigned chemical shifts of C_α_, C_β_, N, H_α,_ and HN. 100 conformers were calculated with Xplor-NIH^35^ using the standard simulated annealing method. 20 conformers with the lowest target function values were selected for analysis.

### Relaxation data analysis

Order parameter (S^2^) and contribution of conformational exchange to transverse relaxation rates (R_ex_) were extracted from R_1_, R_2_, and heteronuclear ^15^N NOE data using the software package DYNAMICS^36^.

### pH titration and pKa calculation

To probe the dependences of chemical shifts on pH, two initial samples each with ~0.5 mM protein were prepared: one in phosphate buffer at pH 7.3 and the other in acetate buffer at pH 5.5. The pH values of the two samples were progressively decreased from 5.5 to 3.5 and from 7.3 to 5.9, respectively, by buffer exchange using centrifugal filter units (Amicon). At each pH value, the ^13^CO chemical shifts were measured from the 2D H(CA)CO spectrum, while ^13^C_δ_ or ^1^H_δ_ shifts were measured from the HSQC spectrum. pKa values of Asp, Glu, and His side-chains were extracted by fitting the titration profiles to the Henderson-Hasselbach equation^37^,


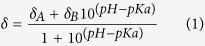


where δ_A_ and δ_B_ are the plateau values of chemical shifts in the acidic and basic pH limits, respectively. The uncertainties in pKa mainly resulted from the error (0.05) in pH measurements were estimated by running 100 Monte Carlo simulations. For each residue 100 profiles were generated by adding random Gaussian noise with a standard deviation of 0.05 to the measured pH values. Subsequently, the 100 profiles were fitted to eq. (1) to obtain a distribution of pKa values.

### Chemical shift perturbation

The combined chemical shift difference of an amide in two samples was calculated by^38^:


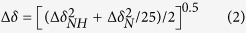


where Δδ_NH_ (Δδ_N_) is the ^1^H_N_ (^15^N) chemical shift difference between pH 7.3 and 5.9.

### F-actin depolymerization assay

Actin purification, pyrene-labeling, and the F-actin severing assay followed previously described procedures^39^ with slight modifications. For F-actin depolymerization, 10 μM gelsolin-capped F-actin (10% pyrene-labeled, gelsolin:actin ratio 1:500) was diluted to a final concentration of 0.5 μM in the absence/presence of 0.5 μM G1+ in buffers at pH 5.0 (50 mM sodium acetate, 45 mM NaCl, 1 mM MgCl_2_, 3 mM EGTA,. 0.2 mM ATP, 0.5 mM TCEP), pH 5.9 (50 mM MES, 45 mM NaCl, 1 mM MgCl_2_, 3 mM EGTA, 0.2 mM ATP, 0.5 mM TCEP), or pH 7.5 (50 mM HEPES, 50 mM KCl, 1 mM MgCl_2_, 1 mM EGTA, 0.2 mM ATP, 0.5 mM DTT). The change in fluorescence intensities was recorded in a PerkinElmer LS 55 spectrophotometer with excitation/emission wavelengths set at 365/407 nm.

### Data availability

The structures of G1+ at pH 5 and 7.3 are available at Protein Data Bank (PDB) and can be accessed through ID numbers 5H3M and 5H3N, respectively. The NMR resonance assignments and distance restraints are available at Biological Magnetic Resonance Bank (BMRB) and can be accessed through BMRB entry numbers 36026 and 36027, respectively.

## Additional Information

**How to cite this article:** Fan, J.-s. *et al*. Structural Basis for pH-mediated Regulation of F-actin Severing by Gelsolin Domain 1. *Sci. Rep.* **7**, 45230; doi: 10.1038/srep45230 (2017).

**Publisher's note:** Springer Nature remains neutral with regard to jurisdictional claims in published maps and institutional affiliations.

## Supplementary Material

Supplementary Information

## Figures and Tables

**Table 1:** pKa values of Asp, Glu and His residues in G1+.

Residue	D50	D61	D66	D84	D96	D109	D110		
pKa	4.8	4.5	3.9	<3.5	<3.5	5.5	4.3		
Residue	E28	E31	E38	E47	E92	E97	E121	E126	E156
pKa	—	—	4.1	<3.5	4.3	<3.5	<3.5	<3.5	3.8
Residue	H29	H86	H119	H151					
pKa	5.9	4.2	4.3	6.2					
