# Supplementary material for: Structural Basis for pH-mediated Regulation of F-actin Severing by Gelsolin Domain 1
Source: Sci Rep. 2017 Mar 28;7:45230. doi: 10.1038/srep45230 (PMC5368644; doi:10.1038/srep45230)
Supplement: Supplementary Information [file srep45230-s1.pdf]

Supplementary Information

Structural Basis for pH-mediated Regulation of F-actin Severing by Gelsolin Domain 1

Jing-song Fan[1], Honzhen Goh[1], Ke Ding[2], Bo Xue[2], Robert C. Robinson[2-6], Daiwen Yang[*,1]

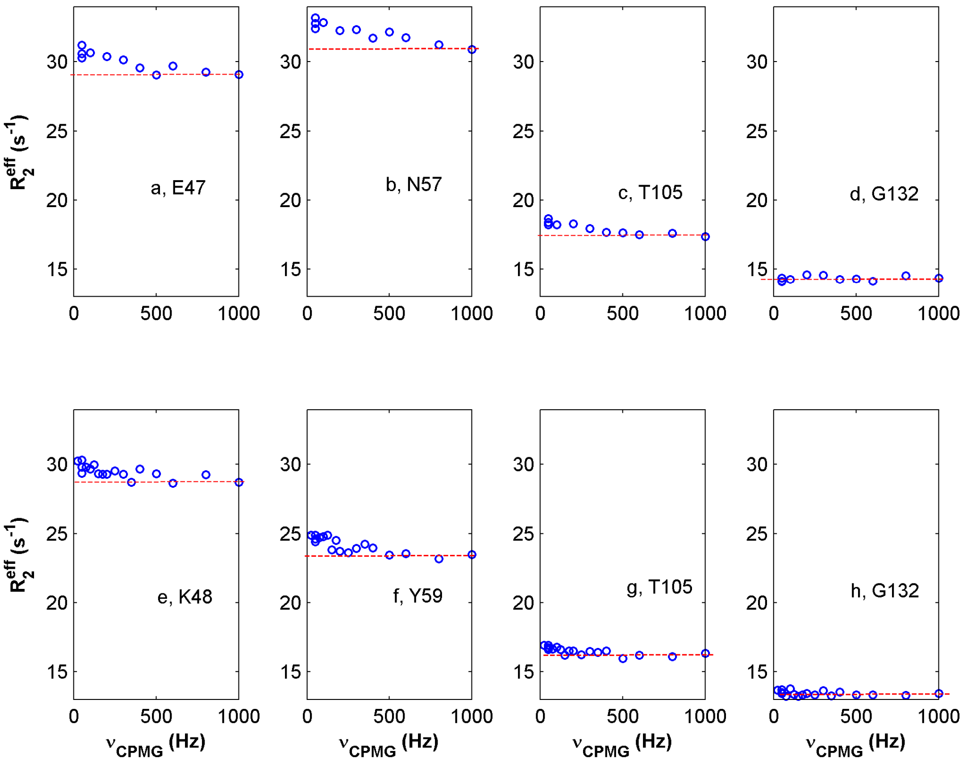

Figure S1. Representative ^{15}N relaxation dispersion profiles of G1+ at pH 5.0 (a-d) and pH 7.3 (e-h), which show the dependences of ^{15}N relaxation rates on CPMG field strengths. Experimental data are indicated by "o". To estimate the uncertainties of R^2_{eff}, the relaxation rates at the ν_{CPMG} field strength of 50 Hz were measured three times. The uncertainties were 1.5%, 1.2%, 1.1% and 0.8% for E47, N57, T105 and G132 at pH 5.0, and 1.7%, 1.0%, 0.9% and 1.1% for K48, Y59, T105 and G132 at pH 7.3. The dashed lines represent the relaxation rates at the maximal ν_{CPMG} field strength of 1000 Hz. If no relaxation dispersion exists, the relaxation rates for a given residue are independent of ν_{CPMG}.

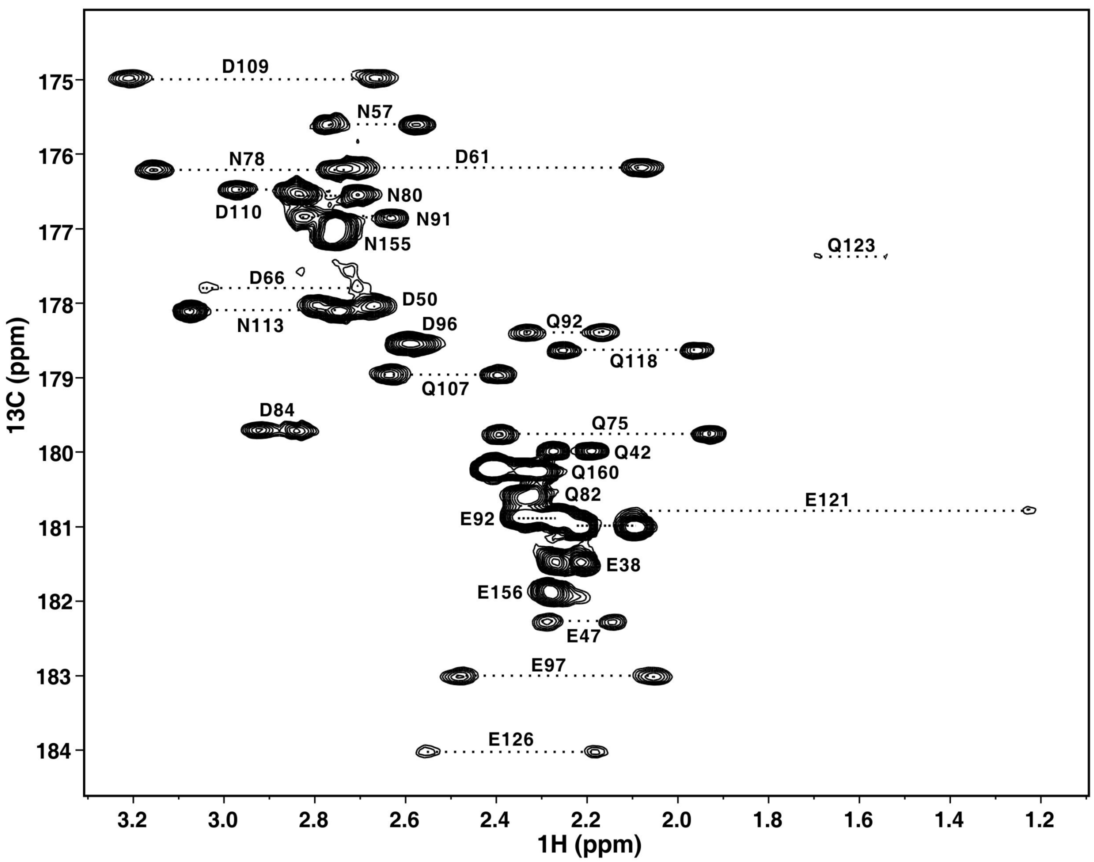

Figure S2. 2D Hβ-CO correlations for Asp (D) and Asn (N) and Hγ-CO correlations for Glu (E) and Gln (Q) of G1+ at pH 3.5. The assigned sidechain correlations are labelled.

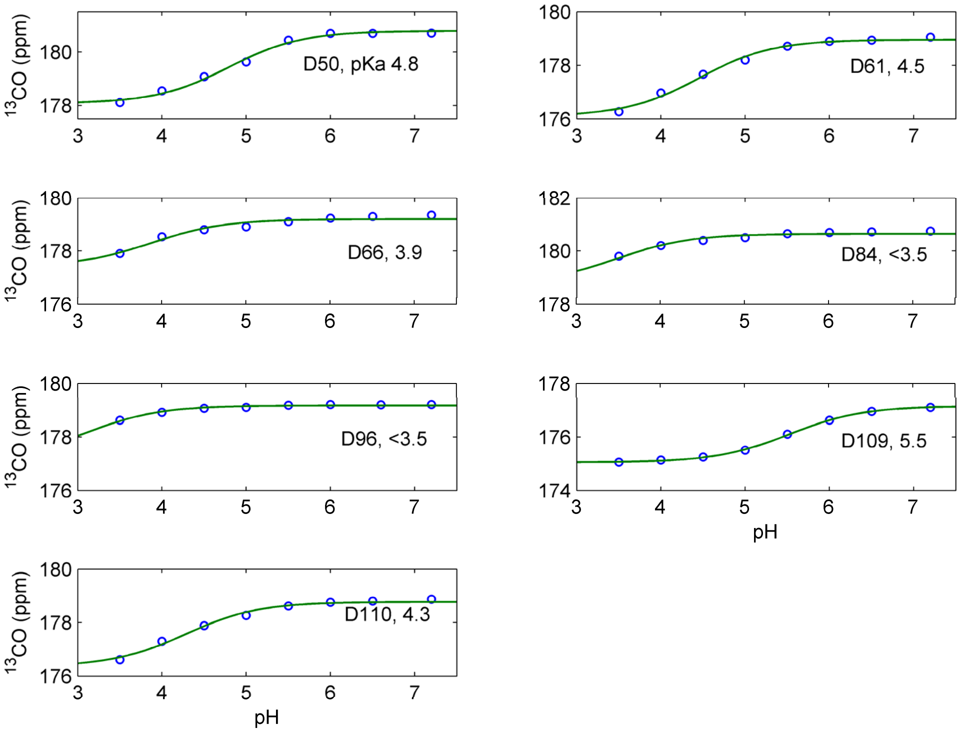

Figure S3. Dependences of ^{13}CO chemical shifts of Asp sidechains on pH. The experimental data are represented by “o”. The solid lines are the best fits. The error in pH was about 0.05.

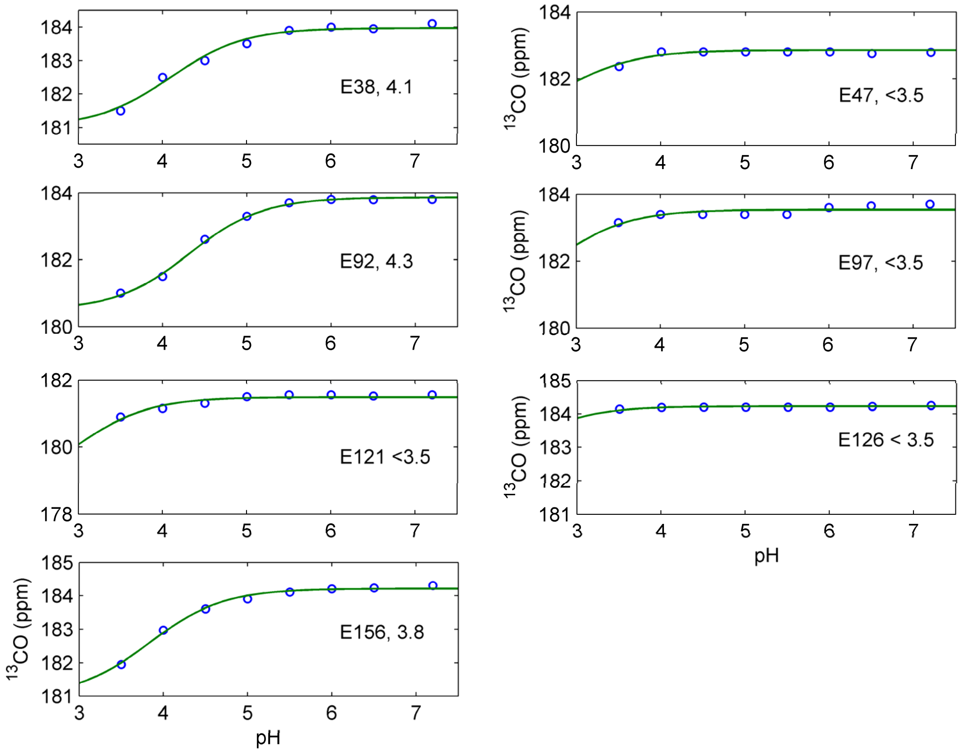

Figure S4. Dependences of ^{13}CO chemical shifts of Glu sidechains on pH. The experimental data are represented by “o”. The solid lines are the best fits. The error in pH was about 0.05.

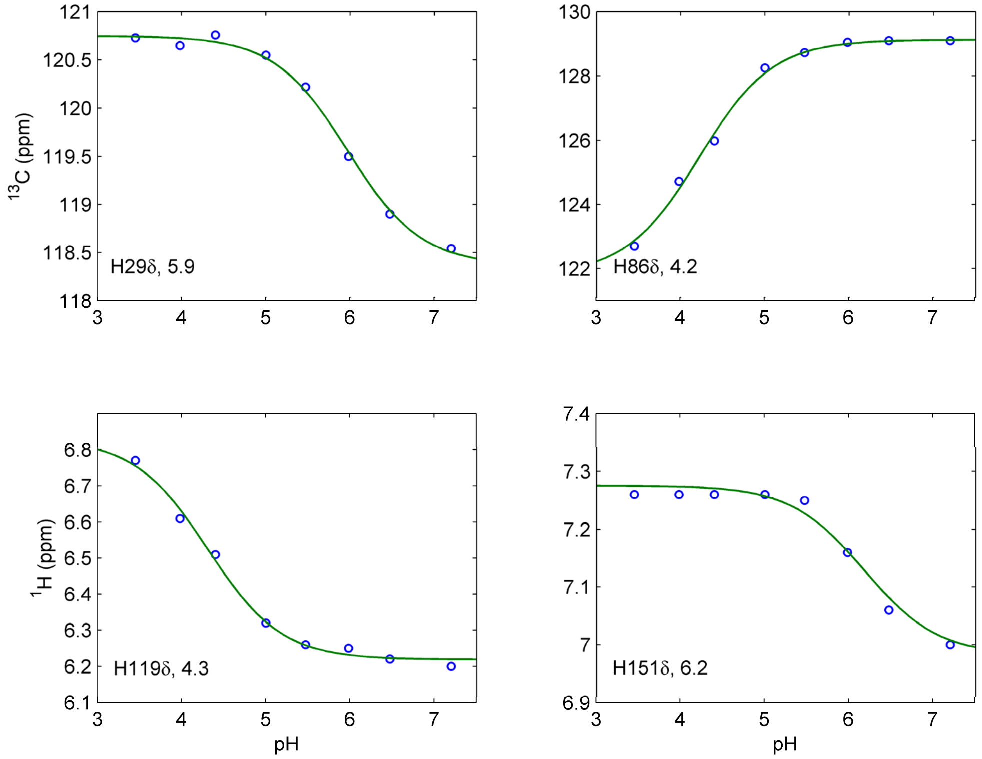

Figure S5. Dependences of $^{13}C_{\delta}$ or $^{1}H_{\delta}$ chemical shifts of His sidechains on pH. The experimental data are represented by "o". The solid lines are the best fits. The error in pH was about 0.05.

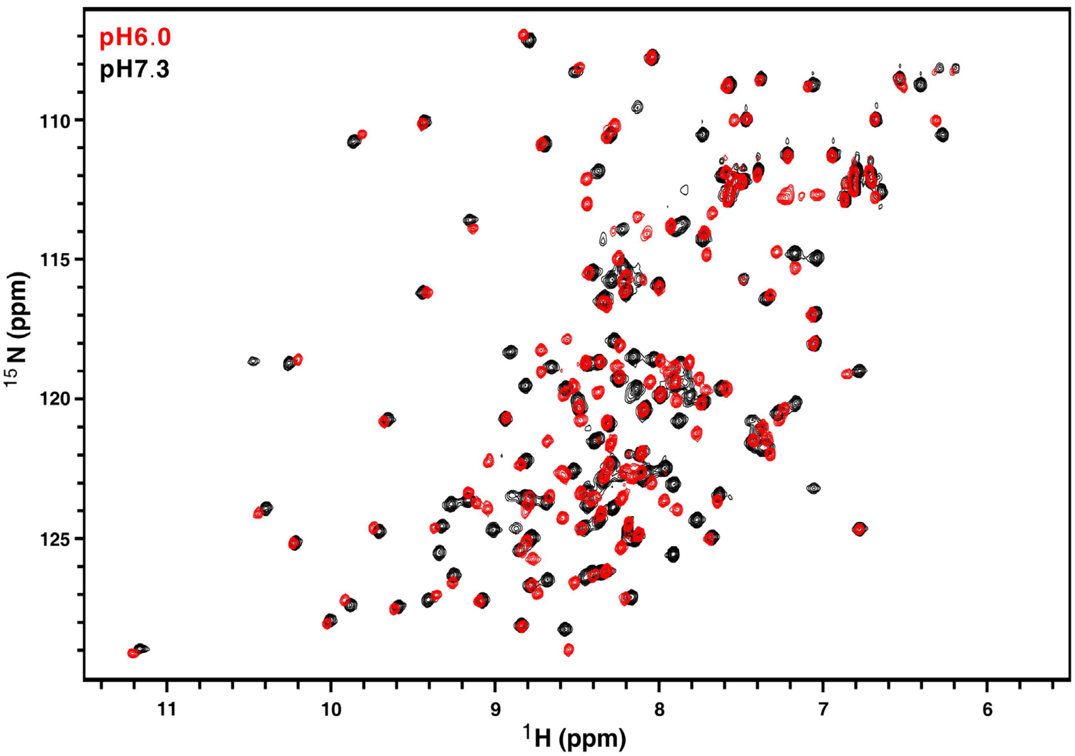

Figure S6. 2D ^{1}H-^{15}N correlations of G1+ at pH 7.3 (black) and 5.9 (red).

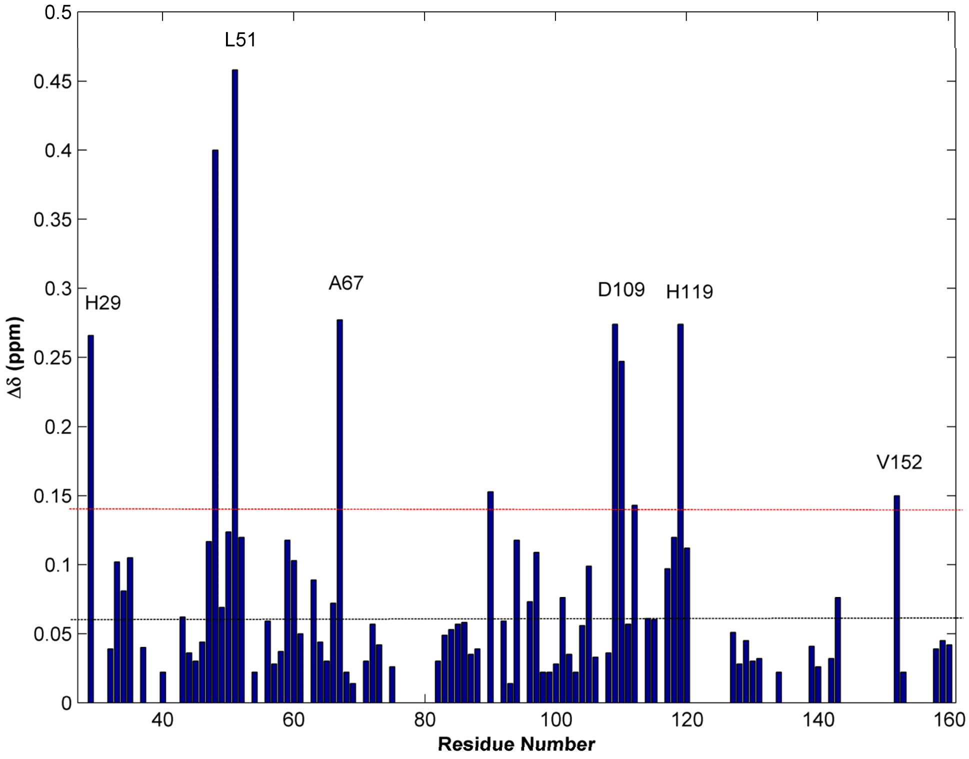

Figure S7. Combined chemical shift differences between pH 5.9 and pH 7.3. The black dashed line represents the average $\Delta\delta$ value over all available residues ($\Delta\delta_{av}$ = 0.06 ppm), while the red dashed line denotes the value of $\Delta\delta_{av}$ + std (0.14), where std is the standard deviation of $\Delta\delta$ values for all available residues.

Table S1. Structural statistics for the final 20 conformers of G1+ at pH 5.0 and pH 7.3

Distance restraints		
	pH 5.0	pH 7.3
Intra-residue (i-j = 0)	302	303
Sequential (|i-j| = 1)	354	310
Medium range (2 ≤ |i-j| ≤ 4)	91	97
Long range (|i-j| ≥ 5)	225	198
Hydrogen bond	84	88
Total	1056	996
Dihedral angle restraints		
ϕ	88	81
ψ	88	81
Average RMSD to the mean structure (Å)[b]		
Backbone atoms	0.87 ± 0.12	0.94 ± 0.17
Heavy atoms	1.64 ± 0.12	1.74 ± 0.15
ϕ/ψ space[c]		
Most favored region (%)	73.2	73.0
Additionally allowed region (%)	19.9	22.5
Generously allowed region (%)	5.5	3.8
Disallowed region (%)	1.4	0.7
rmsd from covalent geometry		
Bonds (Å)	0.001 ± 0.000	0.001 ± 0.000
Angles (deg.)	0.217 ± 0.006	0.221 ± 0.004
Impropers (deg.)	0.191 ± 0.004	0.192 ± 0.004
rmsd from experimental restraints		
NOEs (Å)	0.016 ± 0.001	0.021 ± 0.001
Dihedral angles (deg.)	0.300 ± 0.025	0.289 ± 0.020

[a] Selected from 100 calculated conformers according to overall energy.

[b] Calculated with MOLMOL over secondary structure region (31-34, 40-47, 50-53, 61-63, 67-73, 82-89, 95-112, 117-121, 128-133, 139-141).

[c] Calculated with PROCHECK-NMR.
